# Integrating plant phenotypic and genotypic data in the AGENT project: a BrAPI service implementation

**DOI:** 10.1093/bioinformatics/btag287

**Published:** 2026-05-07

**Authors:** Matthijs Brouwer, Jens Bauernfeind, Gouripriya Davuluri, Jorge García Brizuela, Patrick König, Suman Kumar, Matthias Lange, Stephan Weise, Erik Wijnker, Cyril Pommier, Joseph Ruff, Paul J Kersey

**Affiliations:** Plant Science Group, Wageningen University & Research, WUR, Droevendaalsesteeg 1, 6708 PB Wageningen, The Netherlands; Leibniz Institute of Plant Genetics and Crop Plant Research (IPK), Gatersleben, 06466 Seeland, Germany; Leibniz Institute of Plant Genetics and Crop Plant Research (IPK), Gatersleben, 06466 Seeland, Germany; Leibniz Institute of Plant Genetics and Crop Plant Research (IPK), Gatersleben, 06466 Seeland, Germany; Leibniz Institute of Plant Genetics and Crop Plant Research (IPK), Gatersleben, 06466 Seeland, Germany; Leibniz Institute of Plant Genetics and Crop Plant Research (IPK), Gatersleben, 06466 Seeland, Germany; Leibniz Institute of Plant Genetics and Crop Plant Research (IPK), Gatersleben, 06466 Seeland, Germany; Leibniz Institute of Plant Genetics and Crop Plant Research (IPK), Gatersleben, 06466 Seeland, Germany; Plant Science Group, Wageningen University & Research, WUR, Droevendaalsesteeg 1, 6708 PB Wageningen, The Netherlands; Université Paris-Saclay, INRAE, URGI, 78026 Versailles, France; Royal Botanic Gardens, Kew, Richmond, Surrey, TW9 3AE, United Kingdom; Royal Botanic Gardens, Kew, Richmond, Surrey, TW9 3AE, United Kingdom

## Abstract

**Motivation:**

The AGENT project established a network of actively cooperating European genebanks, integrating genomic and phenotypic data from accessions of wheat and barley. Due to specific storage demands for phenotypic and genotypic data, the project used separate database instances and backend technologies to manage integrated phenotypic and genotypic data.

**Results:**

We discuss the challenges encountered when integrating dispersed data to serve through a single interface such as the Plant Breeding Application Programming Interface, BrAPI. We examine how the consistent mappability of genebank data to the BrAPI model can enable the implementation of effective services. The advantages of BrAPI in transparently linking distributed data entities through embedded, unique identifiers are highlighted. We present a technical solution involving a BrAPI proxy, which combines and merges separate BrAPI endpoints. Finally, we demonstrate the AGENT BrAPI implementation with an illustrative example that validates a suggested SNP for a trait from the literature by linking phenotypic, genotypic and passport data.

**Availability and implementation:**

The BrAPI proxy implementation and documentation is available at the Python Package Index (https://pypi.org/project/brapi-proxy) and archived in Zenodo (doi: 10.5281/zenodo.19436445).

**Supplementary information:**

A Jupyter Notebook file for the validation example using a marker-trait relationship found in the literature.

## 1 Introduction

The Breeding API is an international, widely adopted specification designed to connect information systems and tools for plant breeding-related data through a standardized remote interface and exchange format (Selby *et al.* 2025). Its implementation by major data providers boosts data interoperability, one of the key pillars of the international roadmap for FAIR data. FAIR refers to the principles of Findability, Accessibility, Interoperability, and Reusability, which aim to improve the stewardship, exchange, and long-term reuse of research data (Wilkinson *et al.* 2016). In this context, the EU financed the AGENT project, which focused on the activation of a network of 18 genebanks and research institutes and engineered a suitable research data infrastructure to support the interoperability of phenotypic and genotypic data from wheat and barley. This enabled the implementation of a harmonized data flow (Alaux *et al.* 2024) and the active curation of historic genebank data (Le Floch *et al.* 2026). The curated data was then integrated into domain-specific and data-type-specific optimized information systems. A DivBrowse (König *et al.* 2023) instance was used as backend for efficient query and retrieval of large genomic data, formatted in multidimensional arrays. while passport data and phenotypic data was stored in a relational database management system. The AGENT web portal integrates these datasets for explorative and visual user interaction.

To ensure long-term availability, passport and phenotypic data will be transferred to the European Search Catalogue for Plant Genetic Resources (EURISCO) (Kotni *et al.* 2023), while genotyping data have been submitted to the EMBL ENA and EVA databases.

For machine-actionable access, this separation of data infrastructures raises a significant but common challenge in projects linking diverse data types, thus highlighting a need for a more generally applicable solution for integrated, machine-usable data access.

In any distributed information system, the use of common identifiers across different data sources, such as DOIs assigned to plant genetic resources (Alercia *et al.* 2018), is critical to allow for their integration and joint mobilization. A complication is that data may be generated at different stages in a project’s lifecycle, and some conventions for identifying accessions, samples, and other entities may not be wholly under a project’s control. For example, a sample may need to be managed under an internal identifier prior to submission to a public resource and its assignment of a persistent public identifier. A prerequisite for mastering this complexity is the acceptance by the community of a consistent and well-documented implementation of processes, responsibilities, metadata, data structures and machine-actionable access.

For this purpose, the BrAPI model offers a standardized framework for the management and exchange of breeding data through the mapping of potentially diverse project data in a unified model. This article explores the advantages of BrAPI, the implementation of a BrAPI proxy to merge separate web service endpoints, and a practical demonstration of the AGENT BrAPI implementation.

## 2 Materials and methods

The increased use of genomic information for the management and exploitation of genebank data has been termed “genebank genomics” (Mascher *et al.* 2019). The AGENT project aimed to generate, curate, and integrate passport, phenotypic, and genomic data from various sources, including genebanks, research institutes, and international archives. These data were subsequently used to develop specialized tools for genebank management and analysis. The project used wheat and barley as exemplar crops. Historic and experimental data collected and curated by the project partners were first shared using a project internal instance of the FAIRDOM platform (Wolstencroft *et al.* 2017), which served as a staging area for files prior to validation, curation and upload into backend databases.

Public access was provided through a project-specific web portal, which serves as proof of concept for features that might later be adopted by the EURISCO infrastructure (Kotni *et al.* 2023), that is, the web-based catalogue of the European Cooperative Programme for Plant Genetic Resources (ECPGR). The development was started in the frame of the European Evaluation Network (Kumar *et al.* 2024) and was continued by the AGENT project. It is used in the AGENT project to directly access and query the passport and phenotyping data (Alaux *et al.* 2024). It is the goal of AGENT project partners to migrate much of the data generated in the project into the EURISCO infrastructure, wherever the members of the ECPGR network choose to make AGENT-generated materials (new single seed descent lines from genebank accessions) and their associated data available through EURISCO.

Genotyping data has been submitted to the EMBL European Variation Archive (EMBL-EVA) for long-term archiving as two species-specific submissions. The exchange format is VCF (Danecek *et al.* 2011) and follows the recommendations for the internal formatting of Variant Call Format files to make plant genotyping data FAIR (Beier *et al.* 2022). Within the project, the genotyping data has been made available using separate instances of the graphical variant browser DivBrowse (König *et al.* 2023), which operates directly from the VCF files.

### 2.1 Interfaces

**Figure 1:**
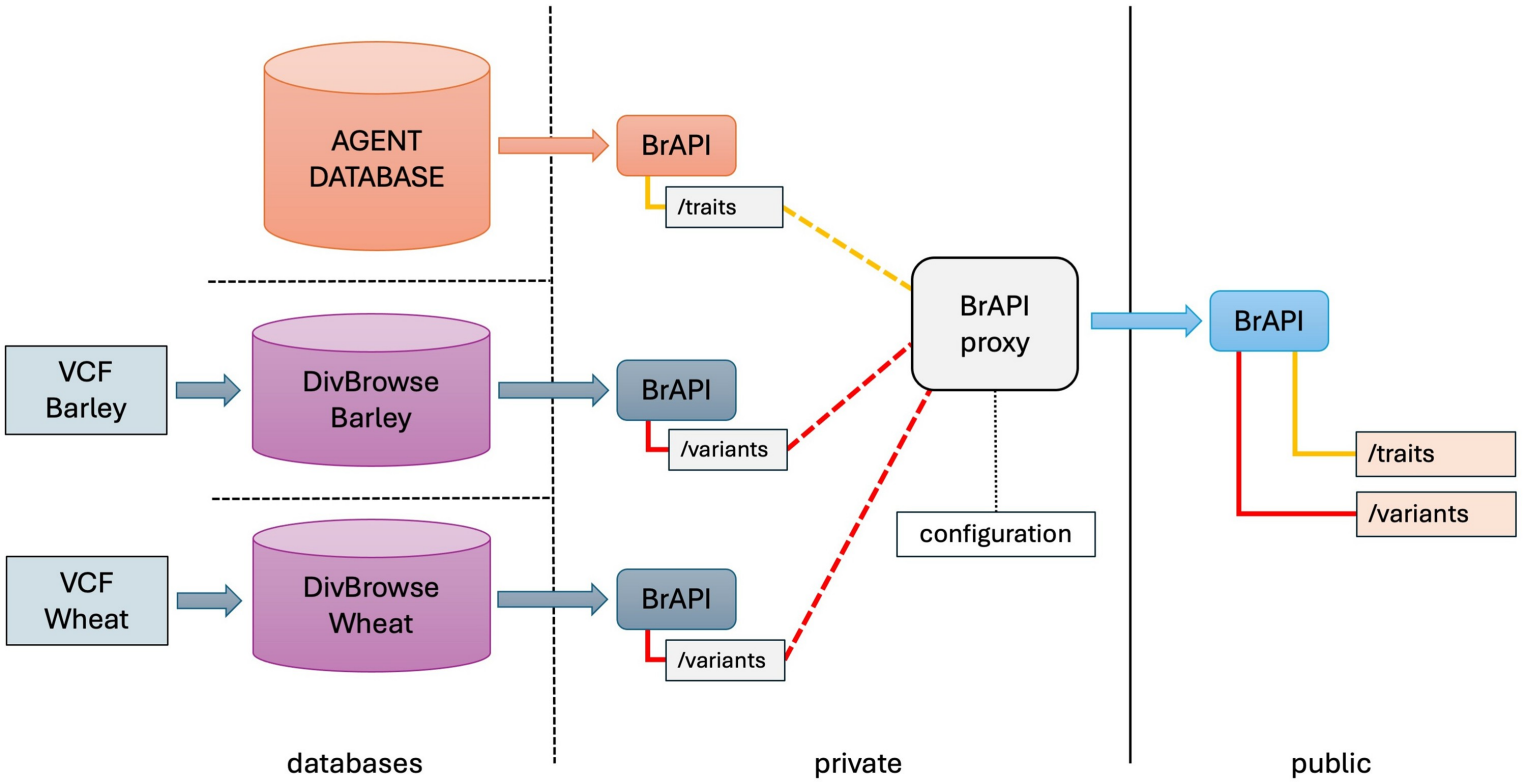
In the AGENT project, we created BrAPI endpoints to publish data distributed across three data sources: the relational AGENT database, which contains passport and phenotypic data, and two DivBrowse instances that hold the genotypic information from VCF files for wheat and barley. For each of these sources, private BrAPI endpoints are available. The developed BrAPI proxy solution merges these private endpoints into public endpoints.

The AGENT portal has been developed to provide an interactive web application to explore and visualize the data from the AGENT database and both DivBrowse instances. The application allows users to search, filter, and visualize the AGENT data via text boxes, menus, and checklists. As a complement, a machine-actionable interface was implemented as RESTful service using the BrAPI specification to enable interoperability with other plant breeding databases and tools. This enables users to automatically fetch, retrieve, and feed AGENT data into downstream analysis pipelines and to combine it with data from other sources that is also available via BrAPI endpoints. BrAPI-compliant endpoints are implemented on the AGENT database to expose the passport and phenotypic data. Both DivBrowse instances expose genotypic data through their own BrAPI-compliant endpoints (Fig. 1).

A significant portion of the effort involved in developing a BrAPI service is mapping the data model of the proprietary project database to the standardized BrAPI data model. Such mapping can be a complex, iterative process, often requiring a feedback loop between developers and scientists. For AGENT, we utilized MIAPPE (Minimum Information About Plant Phenotyping Experiments), an open and community-driven approach that aims at harmonizing data of plant phenotyping experiments (Papoutsoglou *et al.* 2020), to harmonize the two schemas. It comprises both a data model for experimental metadata and a checklist of attributes which may help to adequately describe experiments. The AGENT phenotyping data are collected in MIAPPE-compliant spreadsheets. The BrAPI web service specification is also MIAPPE compliant, and a mapping between MIAPPE sections and BrAPI objects is publicly available and allows the exposure of all MIAPPE metadata through the BrAPI interface. Thus, collecting and organizing project metadata to MIAPPE standards not only supports best practices in data management but also simplifies the subsequent mapping process to the BrAPI model.

### 2.2 BrAPI proxy

To enable performant queries suitable for interactive web frontends, the AGENT project hosted three specialized database instances: two for barley and wheat huge multidimensional variant matrices and one for tabular phenotypic and experimental metadata. Relevant BrAPI endpoints were implemented on the relational AGENT database with passport and phenotyping data and also in both DivBrowse instances (which provide BrAPI endpoints for the barley and wheat genotyping data). Creating a unified BrAPI interface for the AGENT project required these scattered endpoints, hosted at different locations, to be merged in a single combined service. This is especially challenging in the case where reports from multiple system-specific endpoints have to be combined into a single system-agnostic report.

**Figure 2:**
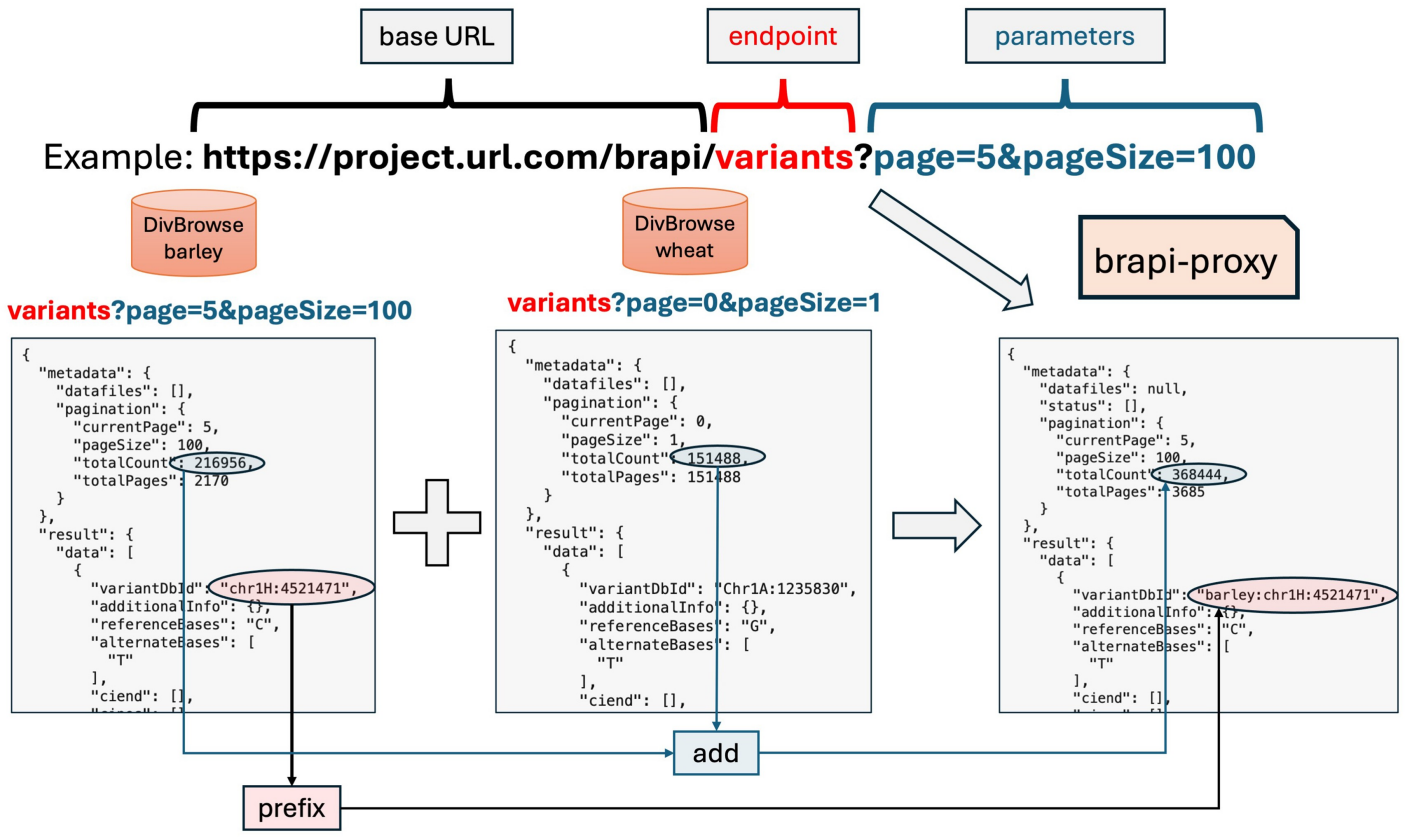
A call to an endpoint provided by the BrAPI proxy solution results in the required calls to underlying endpoints. For most BrAPI endpoints, the response consists mainly of a large list. In case there are multiple underlying endpoints, these responses are merged into a new response. This comes down to recalculating the pagination, selecting the relevant entries from each response list, and where necessary prefix identifiers as configured.

**Figure 3:**
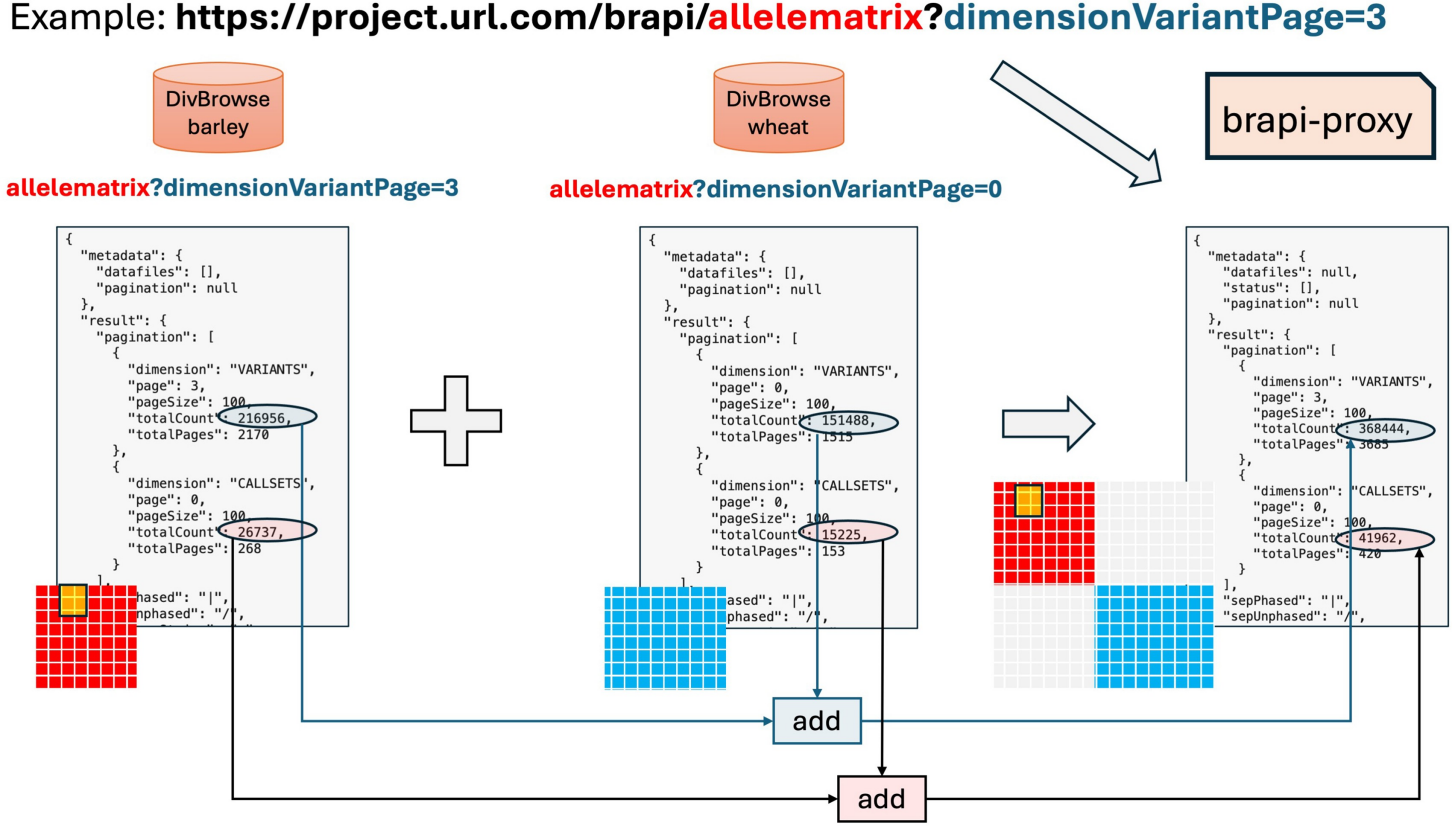
A call to the allele matrix endpoint provided by the BrAPI proxy solution results in the required calls to underlying allele matrix endpoints. Unlike most endpoints, the response here consists of matrices instead of a list, and the corresponding pagination is 2D. In case there are multiple underlying endpoints, this also has to be merged into a new response. This implies recalculating the pagination and merging the matrices.

For most endpoints this involves merging list structures from different locations together and providing an integrated paging system to iterate consistently through the query result (Fig. 2). Merging allele matrix endpoints is more complicated, as this requires combining matrix-structured results and an associated 2D paging functionality (Fig. 3). Moreover, the uniqueness of identifiers used in the different locations has to be maintained in the new combined location. We achieved this by prefixing identifiers from some of the underlying endpoints to avoid ambiguity. Finally, harmonized user authentication and authorization across all the component infrastructures have to be provided. In the AGENT project the partners required the BrAPI interface initially to be restricted to an approved user group prior to the public group.

We developed a customizable BrAPI proxy solution as a template to merge scattered endpoints into a central BrAPI interface and applied this to the AGENT data sources.

## 3 Results

We have shown that a MIAPPE-compliant design of project databases is advantageous for the implementation of a FAIR and machine-usable web service for plant genetic resources data, even on a distributed database infrastructure. A proof is given by the AGENT project, where phenotypic and passport data stored in the AGENT database has been consistently mapped onto the BrAPI data model, and relevant BrAPI endpoints have been realized on this database. For the genotypic data included in the barley and wheat DivBrowse instances, genotypic BrAPI endpoints were provided by this third-party software, which was also designed MIAPPE-compliant. The consequent use of MIAPPE-based record linkage by using a consistent identifier scheme enables machine-actionable access to a network of data records that represent the complex genotype x phenotype relationships across thousands of wheat and barley genotypes. We developed a BrAPI proxy solution able to merge scattered endpoints across backend technology and hosts into a single BrAPI interface for the AGENT project, providing all underlying endpoints and protected by a required authorization header in the requests.

**Figure 4:**
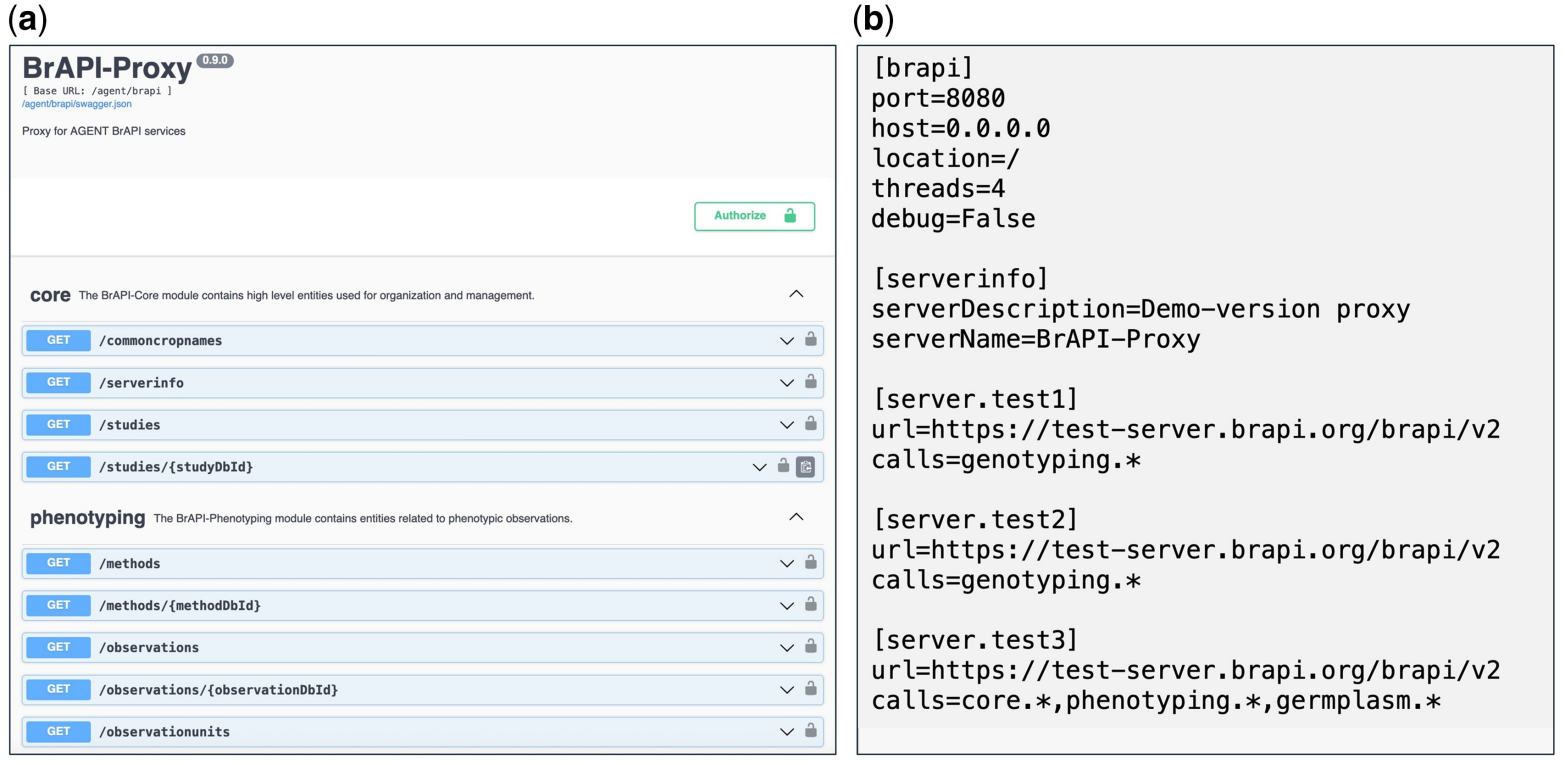
The BrAPI proxy solution has a Swagger UI interface (a) providing an interactive, web-based environment for visualizing and interacting with the API’s resources and operations. The proxy can be configured (b) by simply referring to the location of the underlying endpoints.

The BrAPI proxy is a publicly available and generally applicable Python package. The software provides an easily configurable template to set up a BrAPI server that acts as a broker and forwards requests to configured endpoint URLs. In case of multiple calls, specific URLs are defined for the same endpoint, and the service handles automatically the splitting of requests and merging of responses. Also, the configurable prefixing of identifiers is managed by adding these prefixes for responses and invoking the correct underlying endpoint for requests. A token-based authentication and authorization can be configured individually. Finally, an OpenAPI-compatible API specification for Swagger-featured middleware development is provided (Fig. 4a). For the AGENT project, the configuration of the BrAPI proxy involved referencing the internal BrAPI endpoints on the AGENT database and both DivBrowse instances. To ensure consistency of identifiers used across different locations, crop-specific prefixes were defined for the BrAPI endpoints from both DivBrowse instances.

### 3.1 Validation

In order to validate the implementation and demonstrate the usefulness of the BrAPI interface for the project, we pipelined BrAPI calls to retrieve known marker-trait relations from recent literature (Marzougui *et al.* 2023) on barley, one of the crops of the AGENT project for which phenotypic data was collected from multiple genebanks. Such a relation is found for barley concerning plant height, and we selected the marker with the lowest p-value from this experiment. This is SCRI_RS_154574 on chromosome 6H, position 17007008 bp.

**Figure 5:**
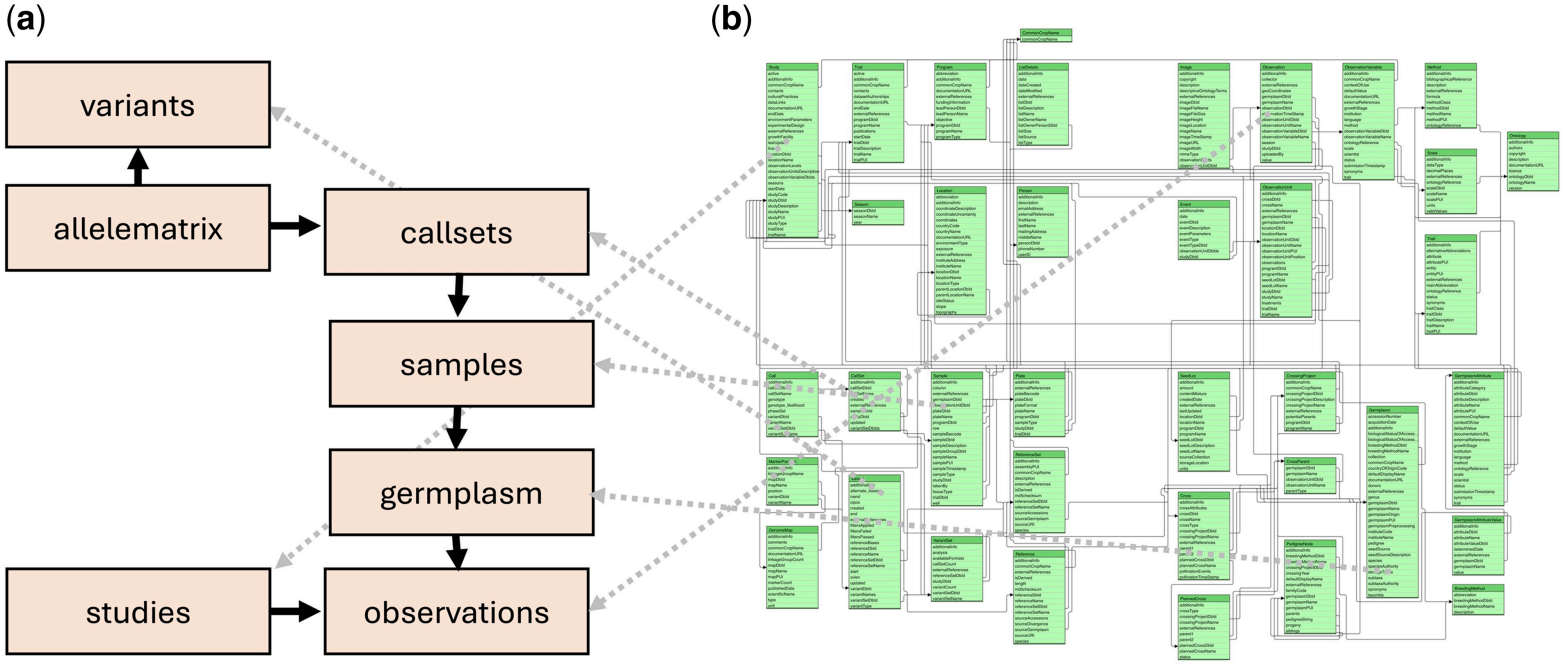
In the BrAPI data model available on brapi.org (b) the available variants are linked by the allele matrix to callsets, samples, germplasm and finally observations. Additional information about the observations may be found in the studies. Each of these entities (a) can be retrieved using the corresponding endpoints.

**Figure 6:**
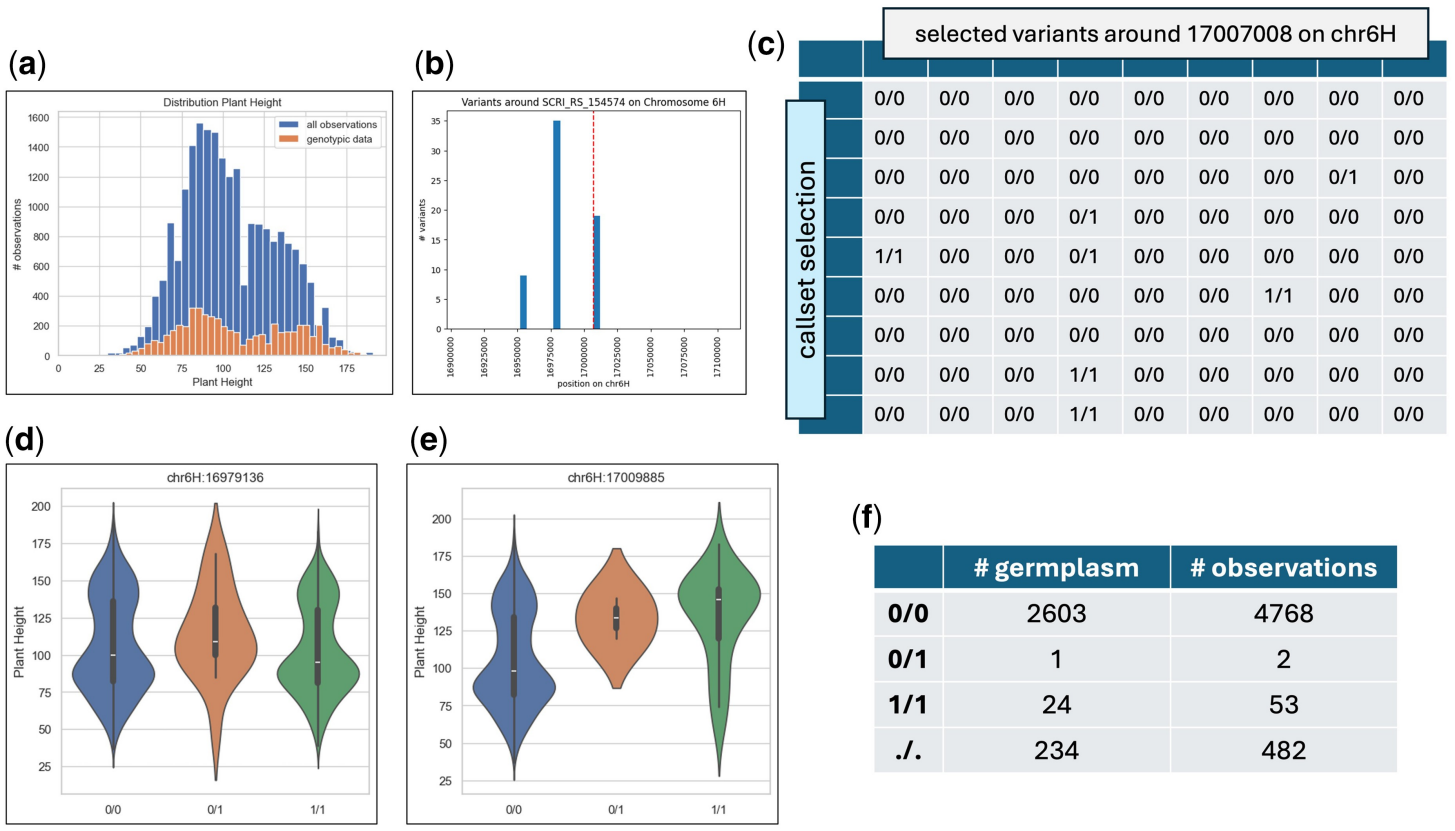
All observations of plant height were collected, and the distribution (a) of these values was computed. Even when restricted to germplasm with available genotypic data, we observe differences in plant height behaviour. Furthermore, variants (b) near a marker suggested by the literature to be related to plant height were selected. For each of these variants, the distribution was computed for each of the values (c) from the alle matrix. For many distributions, no significant differences (d) were observed. However, for the variant at position 17009885 on chromosome 6H (e) we find a confirmation of the suggested marker-trait relation supported by a substantial number of germplasm and observations (f).

To connect this marker to variants within the AGENT project available for barley around this position and compare the trait distributions for different genotypic values, first the relevant BrAPI endpoints have to be identified (Fig. 5). These endpoints correspond directly to the entities within the MIAPPE-compliant BrAPI model onto which the AGENT data has been mapped. Now variants near the position of interest can be selected, and genotypic values can be retrieved by collecting data from the variants and allele matrix endpoints (Fig. 6b). Additionally, all observations of plant height were collected from the observations endpoint and, where possible, linked to callsets from the allele matrix. In the BrAPI model, a identifies a set of calls (genotypes) belonging to a specific sample. The distribution of the plant height, even if restricted to only germplasm for which genotypic data is available, suggests differences in plant height behaviour over observations (Fig. 6a).

**Figure 7:**
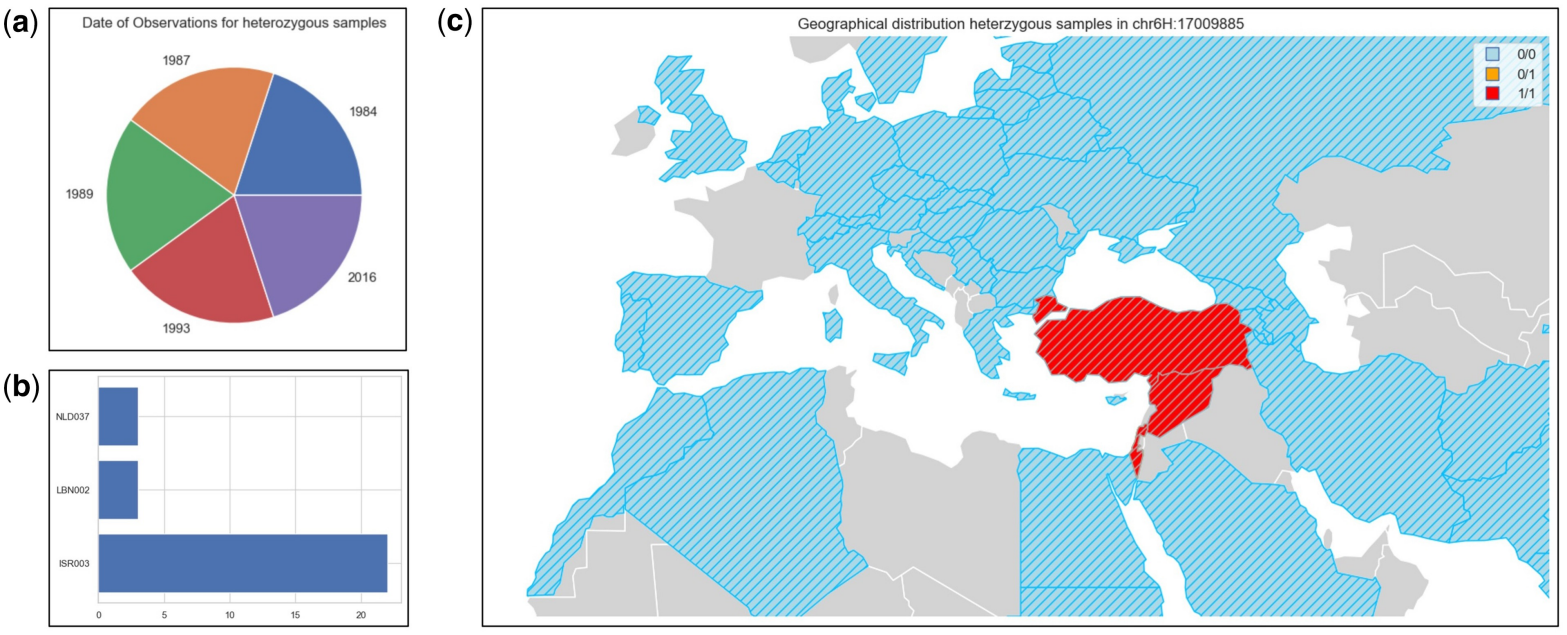
By linking observations for all samples heterozygous or homozygous alternative for the SNP at position 17009885 on chromosome 6H to the studies, the year of observation could be determined (a). From the passport data for these heterozygous or homozygous alternative samples, also the collecting institutes (b) and geographical distribution (c) are known.

By comparing these distributions for the different values from the selected variants, we anticipate confirming the marker-trait relationship identified in the literature. Indeed, for the nearby variant at position 17009885, we observe a significant difference in plant height distribution (Fig. 6e), supported by a substantial number of germplasm and observations. For samples that are heterozygous or homozygous alternative for the identified SNP, we determined the collecting institutes (Fig. 7b), country of origin (Fig. 7c) and year of observation for the plant height (Fig. 7a). Observations were well distributed over multiple years but were geographically clustered.

## 4 Discussion

Publishing isolated data components from a project in a standardized manner through the BrAPI endpoint offers several advantages. First, it enables data retrieval in a uniform and consistent format, enhancing accessibility for software and automated processes. Second, this approach facilitates the connection of related components in a self-describing and FAIR manner. As a result, analysis pipelines from other projects or Breeding API-compatible applications (BrAPPs) can be seamlessly integrated, allowing for more efficient data combination and analysis across diverse sources. This has been demonstrated in the validation of a marker-trait relation from the literature using data from the AGENT project, where harmonized phenotypic, genotypic and passport data from scattered endpoints have been accessed and analysed from only the merged endpoints provided by the BrAPI proxy solution.

In many projects, including the AGENT project, the collection of phenotypic data and the computation of genotypic data are typically managed by different teams and stored in separate databases due to technical and practical considerations. The implementation of the BrAPI service requires mapping all data to the BrAPI model, which facilitates the validation of relationships between potentially distinct stored and constructed data collections, confirming the foreign key relationships between endpoint responses as defined in the model. Additionally, isolated components or inconsistencies in the data can be identified by analysing and comparing the linked responses from the endpoints. However, these aspects are currently not or only very partially covered by the existing BrAPI validator, which primarily focuses on the valid structure of the response (Selby *et al.* 2019). An automated solution to perform the necessary checks would be a valuable and generally applicable tool for both testing the BrAPI service implementation and verifying the mapping and relationships from the underlying internal project databases.

## 5 Conclusion

The implementation of the BrAPI service within AGENT represents a significant advancement in the integration of phenotypic and genotypic data in a machine-actionable way. By mapping project data onto the BrAPI model and utilizing the BrAPI proxy, the project has successfully linked previously isolated data components, thereby improving data accessibility, interoperability and re-usability for researchers. This has been illustrated by an intuitive use case linking passport, phenotyping and genotyping data to validate a marker-trait relationship found in the literature. This example also implicitly validates the relationships among the responses from different BrAPI endpoints. However, introducing a more direct and broadly applicable method for validating these relationships, potentially in the form of a dedicated software package or service that collects and analyses responses from all available endpoints, would significantly enhance the validation process. Such validation could not only test and improve the BrAPI endpoints but also improve the underlying internal project databases, ultimately leading to better data integration and usability.

## Supplementary Material

btag287_Supplementary_Data

## Data Availability

The genomic data used in this study is published through the EMBL European Variation Archive (EVA). The datasets can be found under the following project IDs: PRJEB94114 (Wheat), PRJEB94179 (Barley).
